# Acupuncture and manual therapy for rotator cuff tears: A protocol for systematic review and meta analysis

**DOI:** 10.1097/MD.0000000000020377

**Published:** 2020-05-22

**Authors:** Hongzhi Tang, Fei Luo, Huaying Fan, Li Huang, Shichuan Liao, Wenjing Yu, Yunbei Chen, Xuefei Qin, Jiao Chen

**Affiliations:** a Outpatient department of Sichuan orthopedic hospital; b The Acupuncture and Tuina School, The 3rd Teaching Hospital, Chengdu University of Traditional Chinese Medicine, Chengdu city, Sichuan province, China.

**Keywords:** acupuncture, manual therapy, protocol, rotator cuff tears, Ssystematic review

## Abstract

**Background:**

The tears of rotator cuff is caused by the tears or aseptic inflammation of tendon tissue such as subscapular muscle, supraspinatus muscle, infraspinatus muscle, teres minor muscle, and so on, which make up the rotator cuff. Managements of rotator cuff disease often include acupuncture and manual therapy, usually delivered together. The aim of this review is to assess the effectiveness and safety of such interventions in patients with pain and dysfunction caused by rotator cuff tears.

**Methods:**

We will search the EMBASE, the Cochrane Library, Ovid MEDLINE, PubMed, Web of Science, Chinese Biomedical Literature Database, Chinese National Knowledge Infrastructure, Wanfang Database, the Chongqing VIP, the US National Institute of Health, the NIH clinical registry Clinical Trials, the International Clinical Trials Registry Platform, the Australian New Zealand Clinical Trials Registry and the Chinese clinical registry, from their inception to April 1, 2020. Randomized controlled trials that include patients with rotator cuff tears receiving acupuncture and manual therapy versus a control group will be included. The selection of studies, data extraction and risk of bias assessment will be conducted by 2 independent researchers. A third review author will resolve disagreements. The dichotomous data will be presented as risk ratios with 95% CIs and the continuous data will be presented as weighted mean differences or standardized mean differences with 95% CIs. Evidence quality will be evaluated using the Grading of Recommendations Assessment, Development and Evaluation system.

**Discussion:**

This systematic review will provide updated evidence of various types of acupuncture and manual therapy specifically focuses on its effectiveness and safety for patients’ pain and dysfunction caused by rotator cuff tears.

**Ethics and dissemination:**

Ethical approval is not necessary as this review will not require data from individual patients. The results of this will be published through peer-reviewed journal articles or conference presentations.

**Registration:**

Open Science Framework Preregistration. Registration number 10.17605/OSF.IO/M3NKV.

## Abstract 2

Supplemental Digital Content is available in the text

## 1 Introduction

Rotator cuff tears is one of the common musculoskeletal complaints, and significantly impacts a person's health-related quality of life. Epidemiological studies have reported that 20% of people over 20 years old suffer from rotator cuff tears, while 25% of people over 50 years old suffer from rotator cuff tears, which is the main cause of shoulder joint pain and dysfunction and greatly affects patients’ work efficiency and quality of life.^[1]^ An epidemiological study in the United States reports that 4.5 million people are hospitalized every year for Rotator cuff tears.^[2]^ The rotator cuff is composed of supraspinatus muscle, infraspinatus muscle, subscapularis muscle and teres minor muscle. It starts from the shoulder rib and attaches around the humeral head, forming a sleeve like structure at the anatomical neck of the humeral head. The function of the rotator cuff is to support and stabilize the shoulder and humeral joint, maintain the sealing function of the shoulder joint cavity, maintain the synovial fluid to nourish the articular cartilage, and prevent the secondary osteoarthritis.

The treatment principle of rotator cuff tears is to reduce pain and improve the function of shoulder joint. The most common actions taken at present to relieve the symptoms of rotator cuff tears include steroid injections, oral nonsteroidal anti-inflammatory drugs, physical therapy, exercise therapy, manual therapy, ultrasound therapy and acupuncture.^[3]^ In practice, people with rotator cuff tears seldom receive a single intervention in isolation.^[4,5]^ Acupuncture and manual therapy usually delivered together as components of a physical therapy intervention, are commonly used in the management of rotator cuff tears. Acupuncture is commonly used for musculoskeletal disorders, and it has been suggested as a meaningful nonsurgical intervention for managing shoulder pain and dysfunction.^[6]^ Manual therapy is also commonly used in the management of rotator cuff tears.^[7]^ Manual therapy includes any clinician-applied movement of the joints and other structures, for example mobilization (of which several types exist) or manipulation.

The aims of both types of interventions are to improve function, promote healing, increase joint range, strengthen weakened muscles and correct imbalance in the stabilizing function of the rotator cuff.^[8,9]^ The mechanism of acupuncture treatment has not been fully elucidated. Some studies have pointed out that the mechanism of acupuncture and moxibustion treatment may be related to the local effect, which refers to the release of neuropeptide and other substances by stimulating the sensory nerve endings of skin and muscle tissue. The research shows that acupuncture can promote the release of β-endorphin^[10]^ and increase the concentration of 5-hydroxytryptamine in brain tissue,^[11]^ so as to reduce the sensitivity of tissue to pain and improve joint activity. Manual therapy can stimulate peripheral mechanoreceptors and inhibit nociceptors to reducing pain, and can enhance exchange between synovial fluid and cartilage matrix to increasing joint mobility.^[12]^

Previous systematic reviews have not assessed acupuncture and manual therapy for rotator cuff tears together. Consequently, we decided to conduct a systematic review based on the best currently available evidence and methodology to evaluate whether acupuncture and manual therapy is effect and safety for pain and function of rotator cuff tears.

## 2 Methods

### 2.1 Study registration

Prospective registration of this study has been approved by the Open Science Framework registries (https://osf.io/registries) with a registration number 10.17605/OSF.IO/M3NKV, and the protocol has been written following the Preferred Reporting Items for Systematic Reviews and Meta-Analyses Protocols statement guidelines.^[13]^

### 2.2 Types of studies

We will include randomized controlled trials (RCTs) of acupuncture treatment for pain and dysfunction caused by rotator cuff tears. RCTs in English and Chinese will be included in the review. RCTs using single-blind, double-blind or open-label design will be included. For crossover trials, data are extracted from the first period only, to avoid potential carryover effects.

### 2.3 Types of participants

Adult patients (aged ≥18 years) diagnosed with Rotator cuff tears will be included, with the symptoms of shoulder joint pain or dysfunction enrollment in the research.

We will exclude trials that include any participants with a history of significant trauma or systemic inflammatory conditions such as rheumatoid arthritis, osteoarthritis, hemiplegic shoulders, or pain in the shoulder region as part of a complex myofascial neck/shoulder/arm pain condition.

### 2.4 Types of interventions

All randomized controlled comparisons of acupuncture or manual therapy versus placebo, no treatment, another active intervention will be included. Trials evaluating the primary or add-on effects of acupuncture and manual therapy, acupuncture alone, and manual therapy alone are eligible.

Acupuncture will be considered only of it involves needle insertion at acupuncture points, pain points, or trigger points, and had to be described as acupuncture. We will exclude other methods of stimulating acupuncture points without needle insertion (such as laser stimulation or transcutaneous electrical stimulation). Eligible manual therapy interventions include mobilization, manipulation and massage.

We will exclude studies that only compare different forms or methods of acupuncture (eg, transcutaneous electrical nerve stimulation) and compare acupuncture with any complementary and alternative therapies for which the efficacy is not yet established (eg, Chinese herbs).

### 2.5 Types of outcome measures

No studies were excluded on the basis of outcome measure used.

#### 2.5.1 Primary outcomes

(1) Pain intensity (measured by validated scales, such as the visual analog scale or numeric rating scale);
(2) Shoulder function (measured by validated scales, such as the Constant-Murley score, Shoulder Pain and Disability Index total scale, Dutch Shoulder Disability Questionnaire, Disabilities of the Arm, Shoulder and Hand score, University of California-Los Angeles Shoulder rating scale, or the American Shoulder and Elbow Surgeons rating scale).

#### 2.5.2 Secondary outcomes

(1) Health-related quality of life measured on validated scales such as short-form 36 health survey or EuroQol;
(2) Patient global assessment of treatment outcomes, such as the patient global impression of change;
(3) Adverse events;
(4) Active range of motion of shoulder joint;
(5) Proportion of patients who finally ended up receiving shoulder surgery.

### 2.6 Search methods for identification of studies

#### 2.6.1 Electronic searches

From the inception dates to April 1, 2020, the following databases will be searched: EMBASE, the Cochrane Library, Ovid MEDLINE, PubMed, Web of Science, Chinese Biomedical Literature Database, Chinese National Knowledge Infrastructure, Wanfang Database, the Chongqing VIP. The searching strategy of PubMed is presented in Supplemental Digital Content (Appendix 1).

#### 2.6.2 Searching other resources

Ongoing trials with unpublished data will also be retrieved from the following clinical trial registries: the US National Institute of Health, the NIH clinical registry Clinical Trials. The International Clinical Trials Registry Platform, the Australian New Zealand Clinical Trials Registry and the Chinese clinical registry. Useful but incomplete data will be obtained for data synthesis from the contact trial researcher.

### 2.7 Data collection and analysis

#### 2.7.1 Selection of studies

**Figure 1:**
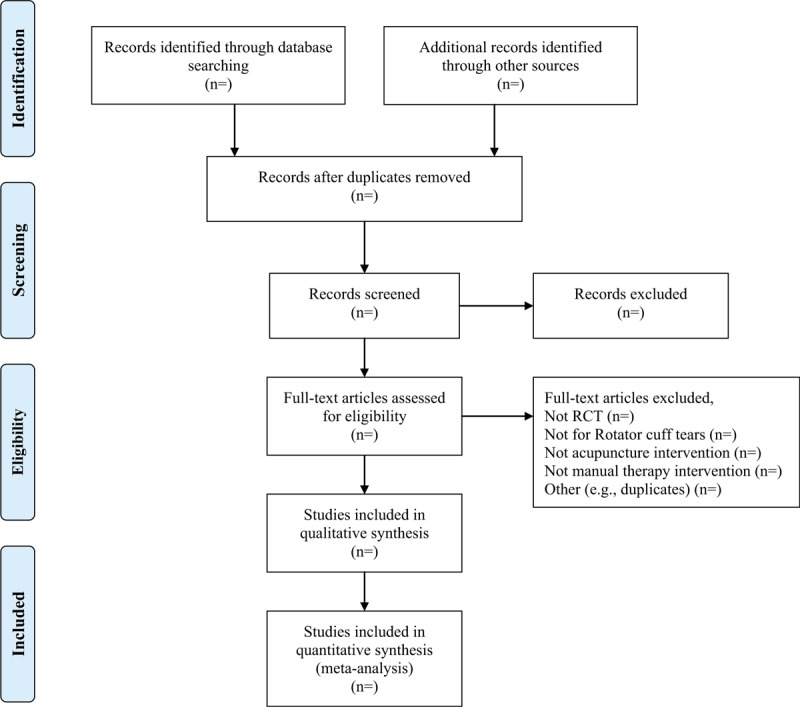
. PRISMA flow diagram of study selection. PRISMA = the preferred reporting items for systematic reviews and meta-analyses.

Two review authors (THZ and LF) will independently screen all abstracts identified from the literature search, then exclude those that are clearly irrelevant (eg, studies focusing on other conditions, reviews, and so on). We will obtain full texts of all remaining references and the same 2 review authors will screen and exclude clearly irrelevant papers according to the selection criteria. We will resolve disagreements by discussion or with a third review author. The detailed process of study selection will be shown in a the Preferred Reporting Items for Systematic Reviews and Meta-Analyses Protocols flow diagram (Fig. 1).

#### 2.7.2 Data extraction and management

Two review authors (THZ and LF) will independently use a specially designed form to extract information on participants, methods, interventions, outcomes, and results. In particular, we will extract first author's name, year of publication, age, sex, duration of disease, sample size, number and type of centers, treated and analyzed, number of reasons for dropouts, duration of baseline, treatment, and follow-up, details of acupuncture treatments (such as selection of points, number, frequency, and duration of sessions), achievement of De-Qi (an irradiating feeling considered to indicate effective needling), number, training, and experience of acupuncturists, details of manual therapy, and the control interventions (sham technique, type, and dosage of drugs). We will contact the first or corresponding authors via email and ask them to provide additional information if necessary.

#### 2.7.3 Assessment of risk of bias in included studies

The quality of the included trials will be evaluated by 2 reviewers using the Cochrane Collaboration's tool.^[14]^ Six aspects (randomly generated sequence number, allocation concealment, blinding of participants and personnel, blinding of outcome assessment, incomplete outcome data, selective reporting, and other bias when required) will be assessed. For each aspect, the trial will be rated as high, low risk, or unclear of bias. A trial that is rated as high risk of bias in 1 or more aspects will be graded as ‘high risk’, while a low risk of bias in all aspects will be graded as ‘low risk’. If there is a low or unclear risk of bias for all main aspects, the trial will be rated as ‘unclear risk’. The contact person or corresponding author will be contacted if basic information is missing for the risk of bias assessment. The rating results will be cross-checked and discrepancies resolved through discussions and the arbitration of a third reviewer.

#### 2.7.4 Measures of treatment effect

Efficacy data will be synthesized and statistically analyzed by using Rev Man software (Review Manager Version 5.3 for Windows, The Nordic Cochrane Centre, Copenhagen). Dichotomous data will be investigated by using a risk ratio with 95% CIs. For continuous outcomes, data will be analyzed by using a standard mean difference with 95% CIs or a weighted mean difference. The weighted mean difference will be used for the same scale or the same assessment instrument; standard mean difference will be used for different assessment tools.

#### 2.7.5 Unit of analysis issues

Different units of analysis will be planned to be subjected to a sensitivity analysis.

#### 2.7.6 Dealing with missing data

We will contact the authors in China via telephone, and authors from elsewhere via email to obtain missing data, if necessary. For all outcomes, we will carry out analyses, and both per-protocol analysis and intention-to-treat analysis will be accepted. Attrition rates, for example dropouts, losses to follow up, and withdrawals, will be investigated. If the missing data is not accessible, we will exclude these articles and synthesis the rest of the included studies.

#### 2.7.7 Assessment of heterogeneity

According to the Cochrane Handbook for Systematic Reviews of Interventions, assessment of between-trial heterogeneity will be based on visual inspection of the forest plot, and more formally on the *I*^2^ statistic. We define that an *I*^2^ of less than 40% is low, 30% to 60% is moderate, 50% to 90% is substantial, and 75% to 100% is considerable.^[14]^

#### 2.7.8 Assessment of reporting bias

We will not perform funnel plots to assess the reporting bias due to the lack of sufficient studies (fewer than 10).

#### 2.7.9 Data synthesis

We will use Rev Man software (Review Manager Version 5.3 for Windows, The Nordic Cochrane Centre, Copenhagen) to carry out statistical analysis. A random effects model will be used to calculate the pooled effect estimates, because substantial clinical heterogeneity is expected among the studies included in this review. If considerable heterogeneity (*I*^*2*^>75%) is observed, we will not meta-analyze the trials and will qualitatively synthesize the data.

#### 2.7.10 Subgroup analysis and investigation of heterogeneity

If 1 of the primary outcome parameters demonstrated statistically significant differences between intervention groups, we will plan to use subgroup analyses. Classifications are as follows:

(1) age of the study population;
(2) type of acupuncture stimulation (ie, manual, electrical, or other stimulation techniques, such as pharmaco-acupuncture, acupotomy, or thread-embedding therapy).

#### 2.7.11 Sensitivity analysis

We will perform sensitivity analyses to determine whether the results have been influenced by trials where radiologic diagnosis of Rotator cuff tears is not mentioned in the participant eligibility criteria, where measures of variance are missing, and where different methods of analysis are used (random-effects model or fixed effect model).

### 2.8 Evidence quality evaluation

Two reviewers will use the Grading of Recommendations Assessment, Development and Evaluation system to independently assess the quality of evidence for each outcome. Evidence quality will be rated ‘high’, ‘moderate’, ‘low’ or ‘very low’ according to the Grading of Recommendations Assessment, Development and Evaluation rating standards. The quality of evidence of a specific study will be assessed according to the risk of bias, inconsistency, indirectness, imprecision, publication bias, large effect, dose-response, and all plausible confounding.^[15]^ A summary of findings table will be generated and included in the final report.

### 2.9 Ethics and dissemination

Ethical approval is not necessary as this review will not require data from individual patients. The results of a review that provide systematically view and evidence of acupuncture and manual therapy for Rotator cuff tears for clinical practice and further research, and it will be disseminated through peer-reviewed journal articles or conference presentations.

## 3 Discussion

Previous systematic studies on shoulder pain have found that acupuncture has insufficient evidence support in improving shoulder joint diseases, partially due to a lack of high-quality primary studies.^[16]^ Effects of manual therapy may be similar to those of glucocorticoid injection and arthroscopic subacromial decompression, but this is also based on low quality evidence.^[7]^ Acupuncture is under consideration as a technique to be applied in Western medical practice for a great many complaints, especially for cases in which modern techniques are either of limited effectiveness or are unsuitable.^[17]^ In addition, the increasing use of acupuncture and manual therapy around the world has not been mentioned in previous reviews, which means that clinical trials and systematic reviews are necessary to assess the effectiveness and safety of such interventions in patients with pain and dysfunction caused by rotator cuff tears. This systematic review would provide updated evidence of various types of acupuncture and manual therapy specifically focuses on its effectiveness and safety for patients with rotator cuff tears.

## Author contributions

THZ, LF, and FHY contributed to the conception and design of the study protocol. The search strategy was developed and run by THZ, CJ, and QXF, who will also screen the title and abstract of the studies after running the search strategy. LF and CYB will screen full copies of the remaining studies after the title and abstract selection. FHY and HL will extract information from the included studies and enter into the electronic database. YWJ will check the accuracy and completeness of the data entry. CJ and HL will give analysis suggestions during data synthesis. All the authors drafted and revised this study protocol and approved it for publication.

## Supplementary Material

Supplemental Digital Content
