# Supplementary material for: Acupuncture and manual therapy for rotator cuff tears: A protocol for systematic review and meta analysis
Source: Medicine (Baltimore). 2020 May 22;99(21):e20377. doi: 10.1097/MD.0000000000020377 (PMC7249925; doi:10.1097/MD.0000000000020377)
Supplement: Supplemental Digital Content [file medi-99-e20377-s001.docx]

| **Appendix 1. Search strategy used in PUBMED database** |
| --- |
| Number Search terms |
| 1 acupuncture therapy  2 manual therapy  3 1 or 2  4 rotator cuff tears  5 rotator cuff disease  6 rotator cuff injury  7 or/4-6  8 randomized controlled trial  9 controlled clinical trial  10 randomized  11 randomly  12 placebo  13 trial  14 or/8-13  15 3 AND 7 AND 14 |
| The search words used in Chinese databases have the same meaning as the version. |
